# Sensitizing Ewing sarcoma to chemo- and radiotherapy by inhibition of the DNA-repair enzymes DNA protein kinase (DNA-PK) and poly-ADP-ribose polymerase (PARP) 1/2

**DOI:** 10.18632/oncotarget.21300

**Published:** 2017-09-28

**Authors:** Britta Vormoor, Yvonne T. Schlosser, Helen Blair, Abhishek Sharma, Sarah Wilkinson, David R. Newell, Nicola Curtin

**Affiliations:** ^1^ Wolfson Childhood Cancer Research Centre, Northern Institute for Cancer Research, Newcastle University, Newcastle upon Tyne, UK; ^2^ Department of Paediatric and Adolescent Haematology and Oncology, Great North Children’s Hospital, Newcastle upon Tyne Hospitals NHS Foundation Trust, Newcastle upon Tyne, UK; ^3^ German Cancer Research Center, DKFZ, Cell Cycle Control and Carcinogenesis, Heidelberg, Germany; ^4^ NORLUX Neuro-Oncology Laboratory, Department of Oncology, Luxembourg Institute of Health, Luxembourg, Luxembourg; ^5^ Northumbria University, Department of Health and Life Sciences, Newcastle upon Tyne, UK; ^6^ Northern Institute for Cancer Research, Newcastle University, Paul O’Gorman Building, Newcastle upon Tyne, UK

**Keywords:** ewing sarcoma, PARP-inhibitor, rucaparib, DNA-PK inhibitor, NU7441

## Abstract

**Background:**

DNA-PK and PARP inhibitors sensitize cancer cells to chemo- and radiotherapy. ETS transcription factors (EWS-FLI1) have been described as biomarkers for PARP-inhibitor sensitivity. Sensitivity to single agent PARP inhibitors has so far been limited to homologous recombination repair (HRR) deficient tumors, exploiting synthetic lethality.

**Results:**

In clonogenic assays, single agent rucaparib LD_50_ values for continuously exposed cells were similar to those observed in HRR-defective cells (CAPAN-1 cell line, BRCA2 defective); however, both ES cell lines (TC-71, CADO-ES1) had functional HRR. *In vivo* rucaparib administration (10 mg/kg daily) showed no responses. In clonogenic assays, rucaparib enhanced temozolomide, camptothecin and radiation cytotoxicity, which was most profound for temozolomide (15–29 fold enhancement). NU7441 increased the cytotoxicity of etoposide, doxorubicin and radiation.

**Materials and Methods:**

We assessed PARP1/2 (rucaparib) and DNA-PK (NU7441) inhibitors in Ewing sarcoma (ES) cell lines by performing growth inhibition and clonogenic assays. HRR was measured by RAD51 focus formation. Single agent rucaparib was assessed in an *in vivo* orthotopic model.

**Conclusions:**

Single agent rucaparib ES sensitivity *in vitro* was not replicated *in vivo*. DNA-PK and PARP inhibitors are good chemo-/radiosensitizers in ES. The future of these inhibitors lies in their combination with chemo-/radiotherapy, which needs to be evaluated in clinical trials.

## INTRODUCTION

The Ewing sarcoma family of tumors (ESFT) is the second most common malignant bone or soft tissue tumor in childhood and adolescence, accounting for approximately 1.5% of all pediatric cancers. Most patients present with localized disease, but up to 25% have metastases at presentation [1]. Significant advances have been made over the past decades with 5-year event-free survival (EFS) rates for patients with localized disease increasing up to 70% [2, 3]. Despite intensive treatment regimes, survival rates for patients with primary metastatic disease have remained poor with 5-year EFS of 39% [2], which is reduced to 13% overall survival in patients with relapsed disease [4]. Better treatment strategies for patients with ESFT are thus an urgent clinical need.

One approach to increasing the efficacy of conventional chemo- or radiotherapy lies in its combination with chemo- or radio-sensitizing agents. Commonly used agents in Ewing sarcoma are the topoisomerase II poisons etoposide and doxorubicin, as well as ionizing radiation, which all cause DNA-double strand breaks (DSB). DSB are one of the most cytotoxic forms of DNA damage and if unrepaired may lead to cell death [5]. Homologous recombination repair (HRR) and non-homologous end joining (NHEJ) repair DSB lesions, with NHEJ being the most important repair pathway in the G0 and G1 phase of the cell cycle [6]. The DNA-dependent protein kinase (DNA-PK) is a central component of NHEJ [7, 8], and a highly potent and specific inhibitor of DNA-PK (NU7441; 2-N-morpholino-8-dibenzothiophenyl-chromen-4-one) has been used successfully in *in vitro* and *in vivo* models to sensitize colon cancer cells and CLL blasts to the effects of DNA-damaging chemo- and/or radiotherapy [9, 10].

Many second line treatment regimens also use topoisomerase I poisons (analogs of camptothecin: topotecan and irinotecan) and the DNA-methylating agent temozolomide that induce DNA single strand breaks. To repair the damage these agents inflict, intact DNA base excision repair (BER) and single strand break repair (SSBR) pathways are required. Poly(ADP-ribose) polymerase 1 (PARP1) is an essential element of SSBR. Inhibitors of PARP1 have been shown to increase the antitumor activity of temozolomide and topotecan in preclinical studies, including models of pediatric cancers [11, 12]. Several PARP inhibitors are in late-stage clinical trial, including combinations with temozolomide and topotecan (reviewed in [13, 14]) and the first study of the combination with temozolomide showed responses in 10/32 patients [15]. However, the most promising clinical utility of PARP inhibitors at present is as single agents in HRR defective tumors, e.g. in BRCA 1 or BRCA 2 defective tumors for which rucaparib recently obtained marketing authorization [16].

Ewing sarcoma (ES) cells are characterized by translocations involving the EWS gene from chromosome 22 and a member of the ETS family of transcription factors, most commonly the FLI1 gene on chromosome 11. Both EWS and EWS-FLI1 proteins interact with BARD1, a putative tumor suppressor, which in turn associates with BRCA1 [17], potentially linking the Ewing sarcoma gene product with HRR. Both PARP1 and DNA-PK interact with EWS-FLI1 [18] and ESFT have high levels of PARP mRNA, protein and polymerase activity [19], and DNA-PK catalytic subunit expression (kids cancer kinome database; http://hgserver1.amc.nl/cgi-bin/r2/main.cgi).

In 2012, cells harboring the EWS-FLI1 translocation have been characterized as being particularly sensitive to PARP-inhibition by a high-throughput screening approach [20], and ES cells and xenografts were sensitive to the PARP-inhibitor olaparib [18]. We wanted to determine whether rucaparib as a single agent is synthetically lethal in ES cells as the EWS-ETS gene product may negatively influence HRR. Additionally we hypothesized that the abundance of PARP and DNA-PKcs implicate a heightened dependence on their activity that might render them particularly sensitive to chemo- and radio-sensitization by PARP or DNA-PK inhibitors.

We report here preclinical data showing that the cytotoxicity of single agent rucaparib was time dependent but *in vivo* experiments failed to demonstrate any measurable effect on tumor growth. The PARP-inhibitor, rucaparib, sensitizes ES cells to temozolomide, camptothecin and ionizing radiation and the DNA-PK-inhibitor NU7441 sensitizes ES cells to chemo- and radiotherapy. Our data strongly support the evaluation of these compounds in combination with chemo- and/or radio-therapy in *in vivo* models and clinical trials.

## RESULTS

### PARP1

#### PARP1 levels and inhibition of PARP1 activity by rucaparib

**Figure 1:**
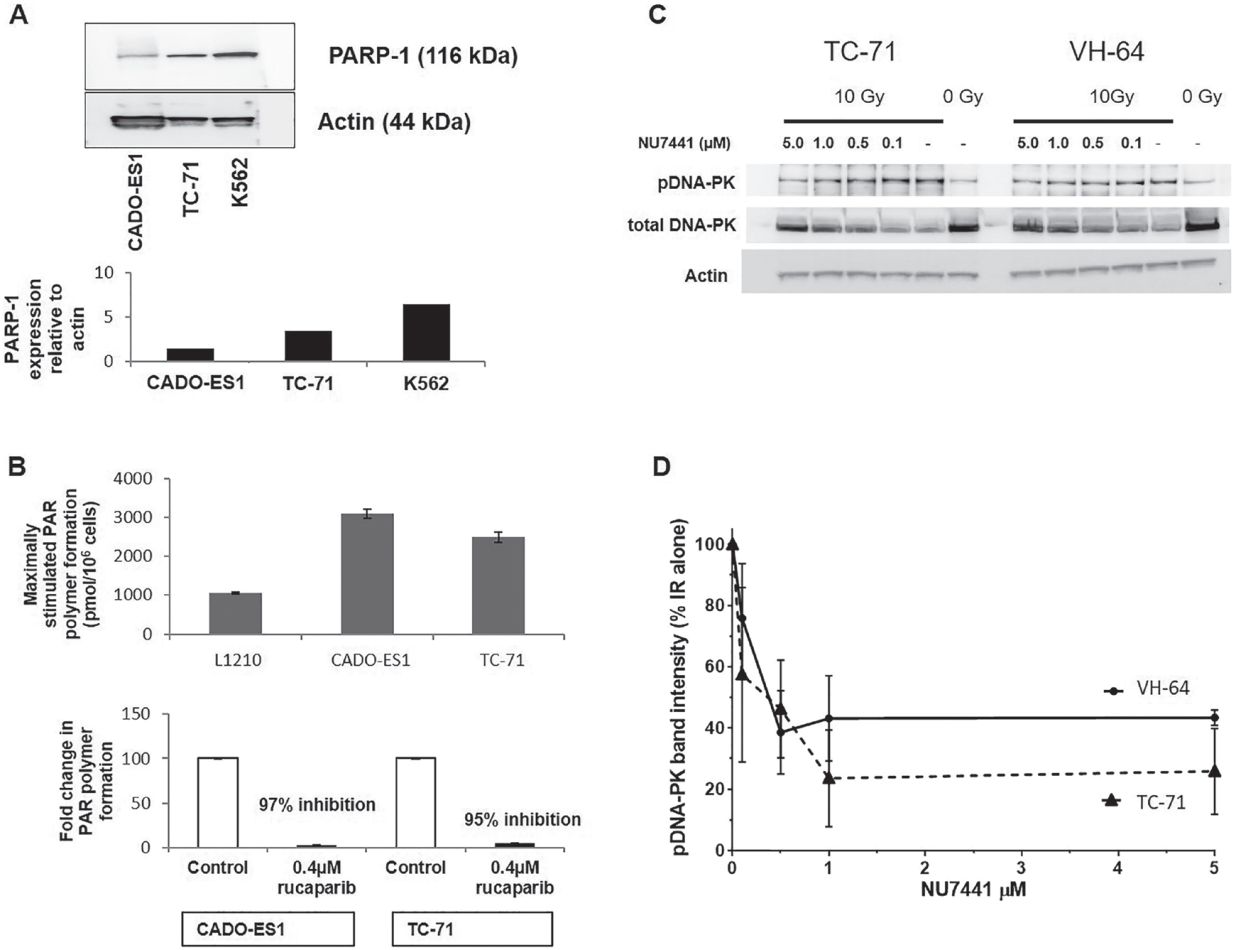
Confirmation of PARP and DNA-PK presence, activity and inhibition by rucaparib or NU7441 (**A**) Western Blot analysis of PARP1 in Cado-ES1, TC-71 and K562 cells. (**B**) PARP activity in CADO-ES1, TC-71 and L1210 cells, and its inhibition by 0.4 μM rucaparib. (**C**) Representative Western Blots for total and phosphorylated DNA-PKcs (pDNA-PK) in TC-71 and VH-64 cells (no ionizing radiation and without NU7441: lanes labelled “0 Gy”), and pDNA-PK signal in response to 10 Gy IR and increasing concentrations of NU7441. (**D**) Densitometric analysis of pDNA-PK levels: data from 3 separate experiments in TC-71 and VH-64 cells after ionizing radiation with 10 Gy, +/− NU7441, depicted are mean values +/− SEM.

PARP1 expression and activity are known to vary widely between cell lines and individuals [21] and this could potentially impact on the response to cytotoxic drugs. We therefore measured PARP1 expression and activity in the ES cells. PARP1 protein was detected in both CADO-ES-1 and TC-71 cells (Figure 1A), with the level of PARP1 in CADO-ES-1 cells being lower than that in TC-71 cells, which in turn was lower than in the reference cell line, K562 (Figure 1A). Despite this difference, both cell lines showed similarly high PARP activity compared to the control cell line L1210 (Figure 1B), and the PARP inhibitor rucaparib at 0.4 µM inhibited activity by > 95% in both cell lines (Figure 1B).

### Single agent rucaparib activity

**Figure 2:**
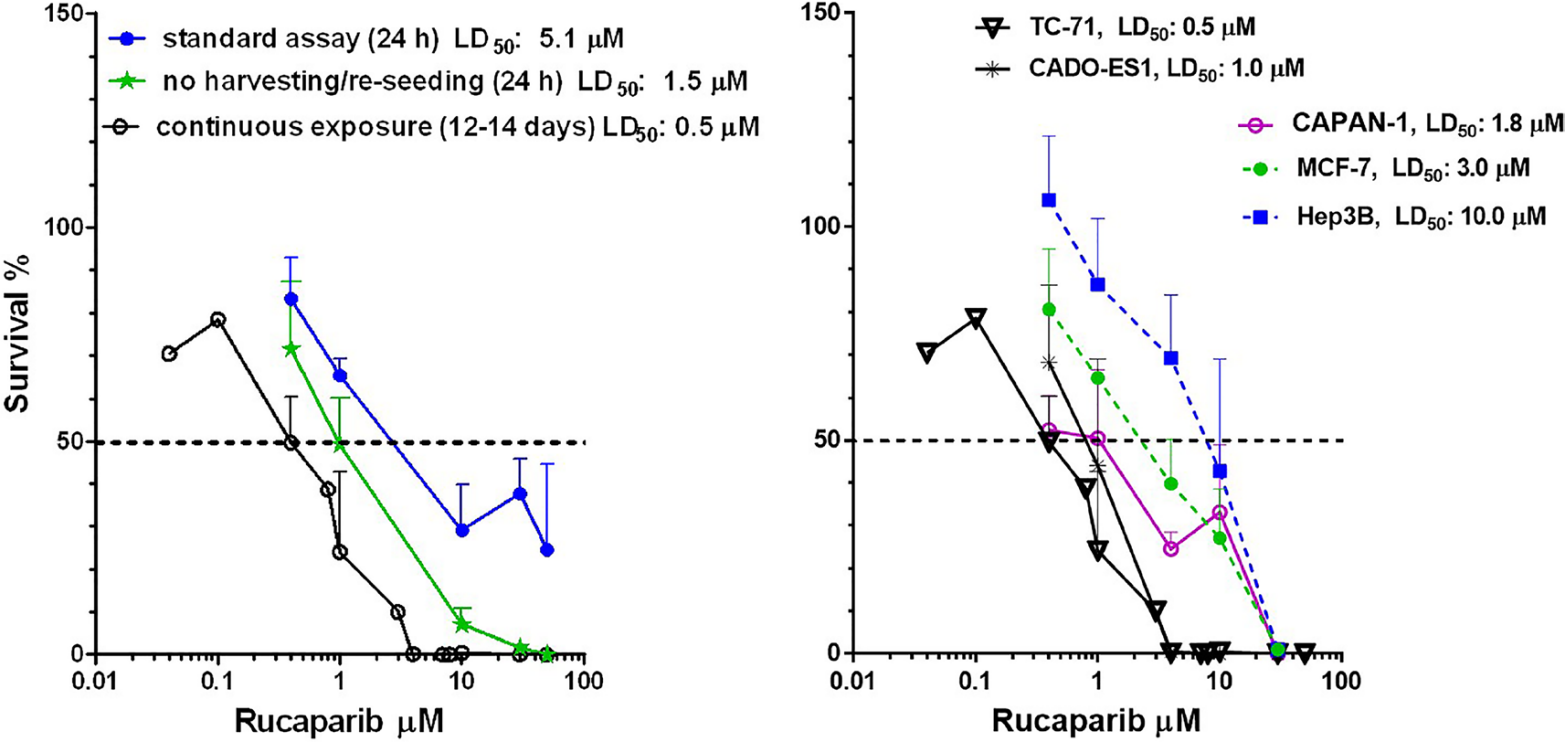
The effect of drug exposure time in clonogenic assays (**A**) Comparison of continuous exposure (12–14 days) to other clonogenic assays (24 h drug exposure with and without harvesting/reseeding) in TC-71 cells. (**B**) Clonogenic assays (continuous exposure) for rucaparib cytotoxicity in 5 different cell lines.

The impact of rucaparib as a single agent on the survival of TC-71 and CADO-ES1 cells was assessed using clonogenic survival assays. The standard assay consisted of a 24 h period of drug exposure, followed by harvesting and re-seeding for colony formation in drug-free medium. In the standard assays, the ES cell lines TC-71 and CADO-ES1 showed similar sensitivities to single agent rucaparib to that observed in growth inhibition assays, with LD_50_ values of 5.1 and 8.0 μM, respectively (Figure 2, left panel for TC-71 cell line). Since the PARP inhibitor cytotoxicity assays in ES reported in the literature [18, 20] involved continuous drug exposure for the duration of the experiment, clonogenic assays were also performed with re-drugging of rucaparib every 3–5 days. In the experiments involving continuous drug exposure, both cell lines were significantly more sensitive to rucaparib than in our standard 24 h exposure assays, with LD_50_ values of 0.5 μM for TC-71 cells and LD_50_ of 1.0 μM for CADO-ES1 cells (Figure 2, right panel). HRR competent control cell lines (MCF-7, Hep3B) were less sensitive to rucaparib (LD_50_ of 3 and 10 μM, respectively). As expected, the HRR defective (BRCA mutant) cell line CAPAN-1 was sensitive towards PARP-inhibition, with a LD_50_ of 1.8 μM.

Rucaparib exposure for 24 h without harvesting/re-seeding of TC-71 cells resulted in intermediate sensitivity to rucaparib compared to either the standard or continuous exposure clonogenic experiments (LD_50_ 1.5 μM) (Figure 2, left panel).

### Ewing cells are competent for homologous recombination repair (HRR)

One possible explanation for the sensitivity of ES cells to rucaparib could lie in the interaction of EWS-FLI1 gene products with BARD1 and its association with BRCA1 [17], which is essential for functional HRR. The HRR status of the ES cells was therefore explored.

**Figure 3:**
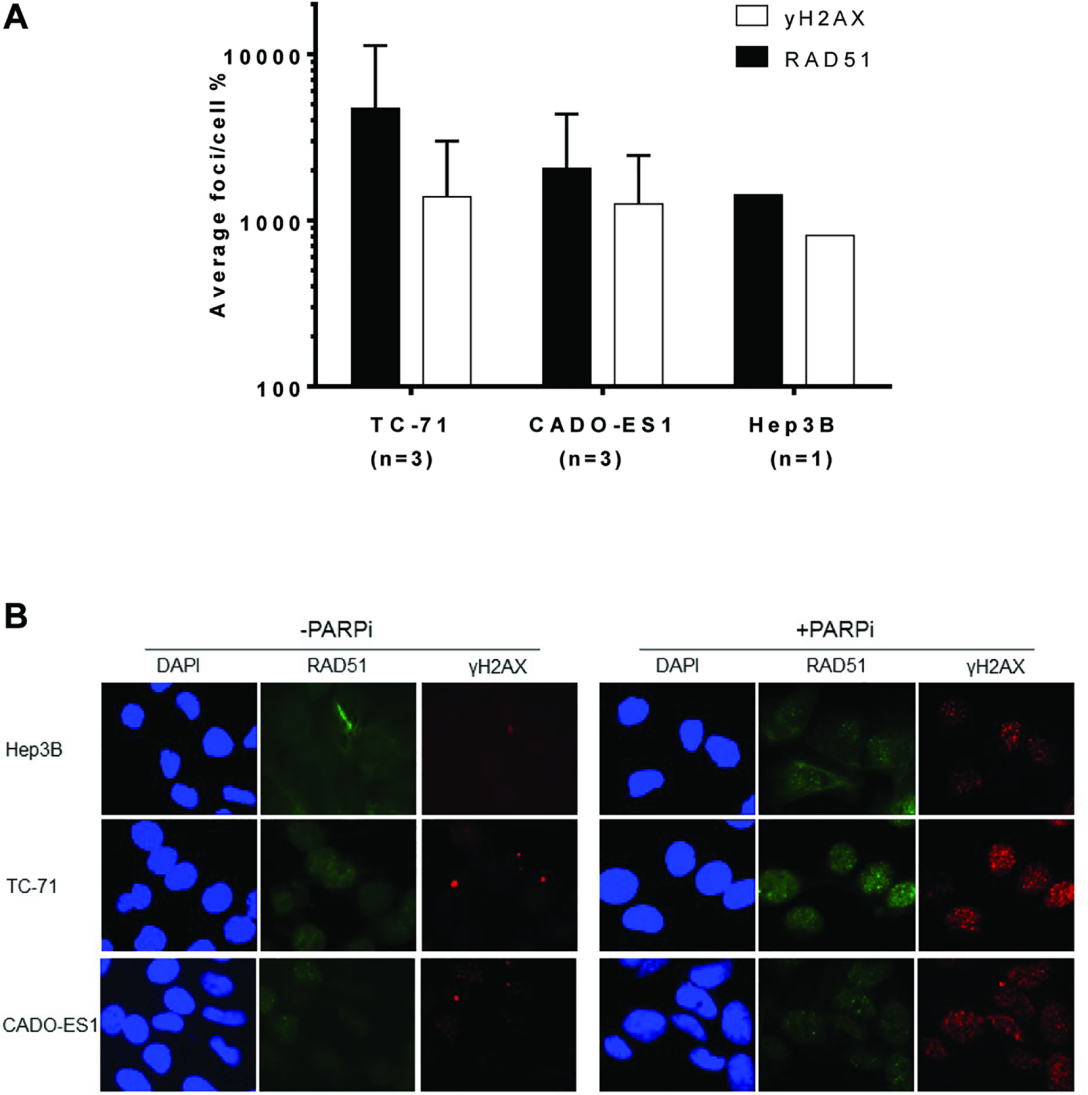
The Ewing sarcoma cell lines TC-71 and CADO-ES1 are competent for homologous recombination repair (**A**) Increase of γH2AX and rad51 foci after 24 h exposure to 10 μM rucaparib, relative to untreated control cells (100%), data for TC-71 and CADO-ES1 cells with the mean +/– SD of 3 repeated experiments. (**B**) Representative microscopy images for data shown in 3A.

HRR function was determined in TC-71 and CADO-ES1 cells with Hep3B cells as a positive control for functional HRR by measuring γH2AX and RAD51 focus formation after 24 h exposure to 10 μM rucaparib. All 3 cell lines showed a significant increase in γH2AX focus formation, indicating the appearance of DSB after inhibition of PARP (Figure 3A). The increase in γH2AX focus formation was more than 10-fold in both ES cell lines, and about 8-fold in the control cell line Hep3B. Equally, all 3 cell lines showed a strong increase in RAD51 focus formation by at least 10-fold, thus there is no impairment of HRR in either cell line (Figure 3A, representative microscopy images Figure 3B).

### Rucaparib as single agent shows no *in vivo* activity against TC-71 tumors

To determine if the cytotoxicity of rucaparib in cell culture experiments translated into an *in vivo* antitumor effect, immunocompromised mice implanted intrafemorally with TC-71 cells were treated with rucaparib.

**Figure 4:**
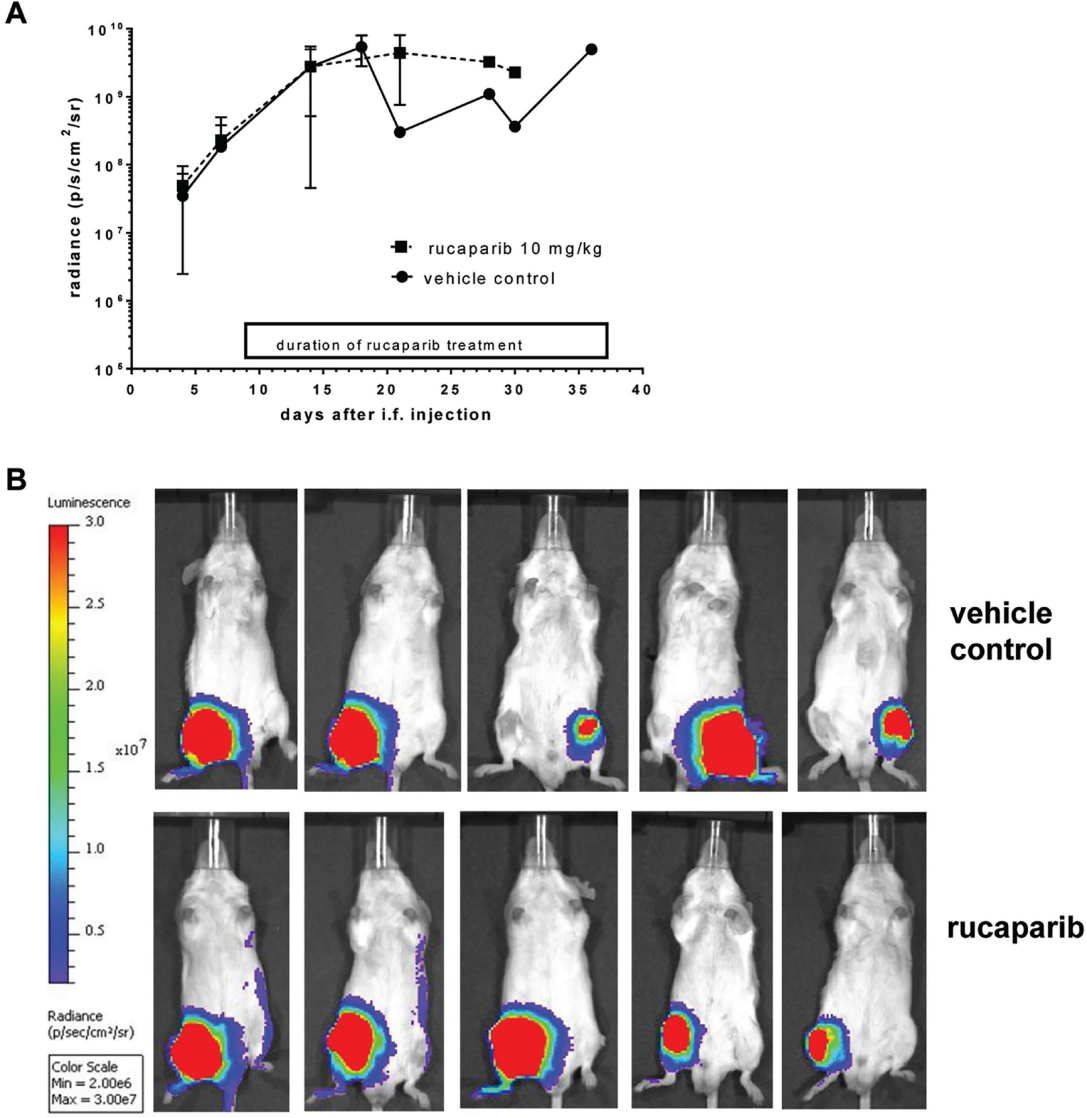
Results of single agent rucaparib in an orthotopic mouse model with TC-71 derived tumors (**A**) TC-71 tumor growth as measured by radiance (p/s/cm^2^/sr). (**B**) Final bioluminescent imaging of each mouse on the respective day of culling.

Mice treated with vehicle control (*n* = 5) or single agent rucaparib (*n* = 5, rucaparib 10 mg/kg daily on 5/7 days for 6 weeks or until the end point for experiment was reached) demonstrated comparable tumor growth characteristics, without any evidence of tumor responses (Figure 4A, 4B). There was no significant clinical toxicity of single agent rucaparib at this dose.

Weight changes of individual mice ranged between −9.5% to +2% total body weight for control animals, compared to −7.4% to −0.3% for rucaparib treated animals (data not shown).

### Growth inhibition assays using rucaparib

Rucaparib on its own did not cause any significant growth inhibition at 0.4 µM; the mean rucaparib GI_50_ concentration for TC-71 cells was 5.0 µM and for CADO-ES1 cells 4.7 µM, i.e. similar or slightly less sensitive than the confirmed HRR-competent MCF7 cell line (GI_50_ 2.4 µM) (Supplementary Figure 1).

TC-71 and CADO-ES1 cells were equisensitive to camptothecin (GI_50_: 3 and 2 nM), ionizing radiation (GI_50_: 2.2 and 1.8 Gy) and temozolomide (GI_50_: 0.3 and 0.25 mM), respectively. Rucaparib at a concentration of 0.4 µM enhanced the cytotoxicity of temozolomide between 5- to 10-fold, of camptothecin 1.4- to 2-fold and ionizing radiation 1.4 fold (Supplementary Figure 2).

### Cytotoxicity assays using rucaparib

#### Chemo-/radio-potentiation

To determine if the reduced cell numbers observed in growth inhibition assays was due to cytotoxicity rather than mere cytostasis, we performed colony formation (clonogenic) assays.

**Figure 5:**
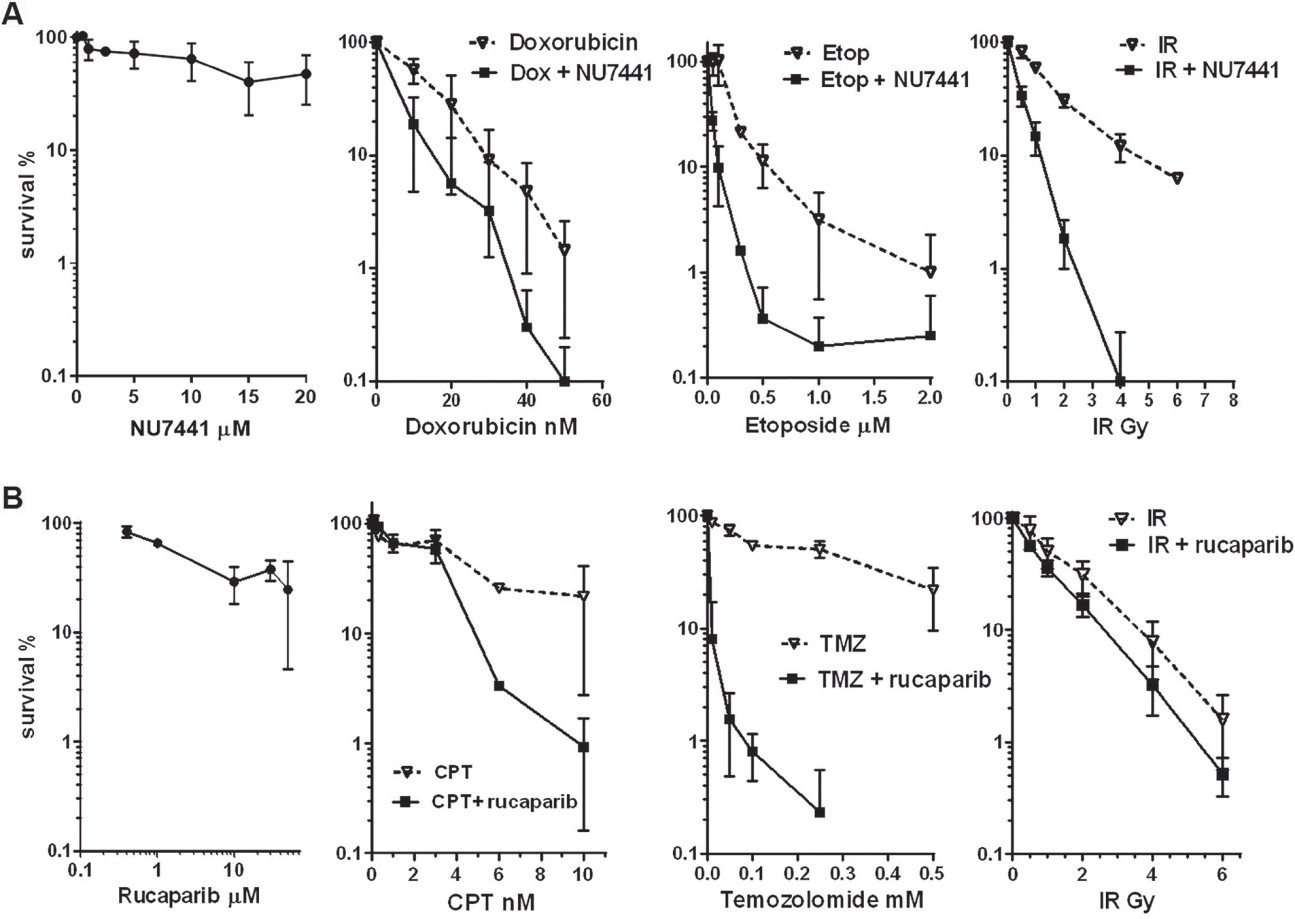
Representative graphs of clonogenic assays in TC-71 cells using NU7441 or rucaparib (**A**) Representative clonogenic assays with NU7441 alone (24 h standard exposure) and 1.0 μM NU7441 in combination with doxorubicin, etoposide or ionizing radiation (IR). Points, mean of triplicate samples from three independent experiments; bars, SD. (**B**) Representative clonogenic assays with rucaparib alone (24 h standard exposure) and 0.4 μM rucaparib in combination with ionizing radiation, temozolomide and camptothecin. Points, mean of triplicate samples from three or four independent experiments; bars, SD.

Both TC-71 and CADO-ES1 cells were approximately equally sensitive to temozolomide with a LD_50_ of 0.28 mM (+/− 0.09 SEM) in TC-71 cells and 0.33 mM (+/− 0.07 SEM) in CADO-ES1 cells. Rucaparib at a non-toxic concentration of 0.4 μM caused a very profound sensitization in both cell lines, with a 29 -fold (+/− 9 SEM) sensitization in TC-71 cells (mean LD_50_: 0.01 mM) and a 15 -fold (+/− 3 SEM) sensitization in CADO-ES1 cells (mean LD_50_: 0.02 mM), all results being highly statistically significant (2-way ANOVA: *p* < 0.0001) (Table 1; Figure 5B).

**Table 1:** Summary of clonogenic assay results

Cell line	Treatment	Clonogenic assays performed (*n*)	Mean LD_50_ (+/–SEM)	Mean DRF_50_ (+/–SEM)	2-way ANOVA
**VH-64**	Doxorubicin Doxorubicin + NU7441	4	12.2 nM (1.3) 5.5 nM (0.26)	2.2 (0.2)	*P <* 0.0001
	Etoposide Etoposide + NU7441	3	0.10 μM (0.01) 0.04 μM (0.01)	2.7 (0.4)	*P <* 0.0001
	IR IR + NU7441	3	0.99 Gy (0.24) 0.36 Gy (0.02)	2.8 (0.7)	*P <* 0.0001
**TC-71**	Doxorubicin Doxorubicin + NU7441	3	13.4 nM (2.3) 6.3 nM (0.6)	2.1 (0.2)	*P =* 0.0008
	Etoposide Etoposide + NU7441	3	0.28 μM (0.05) 0.04 μM (0.01)	6.7 (0.8)	*P <* 0.0001
	IR IR + NU7441	3	1.32 Gy (0.07) 0.39 Gy (0.02)	3.4 (0.2)	*P <* 0.0001
	Temozolomide Temozolomide + rucaparib	3	0.28 (0.09) 0.01 (0.00)	29 (9)	*P <* 0.0001
	Camptothecin Camptothecin + rucaparib	3	5.5 nM (0.6) 4.1 nM (0.8)	1.4 (0.3)	*P =* 0.2
	Ionizing radiation Ionizing radiation + rucaparib	4	1.08 Gy (0.24) 0.68 Gy (0.07)	1.7 (0.4)	*P =* 0.002
**CADO-ES1**	Temozolomide Temozolomide +rucaparib	3	0.33 mM (0.07) 0.02 mM (0.003)	15 (3)	*P <* 0.0001
	Camptothecin Camptothecin + rucaparib	2	6.0 nM (1.3) 2.9 nM (0.3)	2.0 (0.2)	*P =* 0.02
	Ionizing radiation Ionizing radiation + rucaparib	3	1.06 Gy (0.47) 0.74 Gy (0.31)	1.4 (0.1)	*P =* 0.08

LD_50_: concentration of drug necessary to inhibit colony formation by 50%. SEM = Standard Error of Mean. DRF = dose reduction factor, calculated by dividing the LD_50_ results for the cells treated with cytotoxic alone and those of the corresponding cells treated with cytotoxic plus inhibitor.

Summary of all clonogenic assays in Ewing sarcoma cell lines evaluated for combinations of NU7441 (1.0 μM) or rucaparib (0.4 μM) with chemotherapeutic agents or ionizing radiation.

Both cell lines displayed similar sensitivities to camptothecin, with a mean LD_50_ of 5.5 nM in TC-71 cells and 6.0 nM in CADO-ES1 cells. Rucaparib caused a 1.4-fold potentiation (LD_50_ = 4.1 nM) in TC-71 cells and a 2-fold potentiation (LD_50_ = 2.9 nM) in CADO-ES1 cells, but these effects were not, or marginally, statistically significant (*p =* 0.2 and 0.02).

The two cell lines were equally sensitive to ionizing radiation, with LD_50_ values of 1.1 Gy for both TC-71 and CADO-ES1 cells. Rucaparib caused a modest radiosensitization of 1.7 -fold in TC-71 cells (mean LD_50_ 0.7 Gy; *p =* 0.002) and of 1.4-fold in CADO-ES1 cells (mean LD_50_ 0.7 Gy, *p =* 0.08). All of the rucaparib combination data are summarized in Table 1.

### DNA-PK

### DNA-PKcs as a determinant of the effects of cytotoxic treatment

To determine which classes of cytotoxic drugs are dependent on DNA-PK activity for repair of the damage they inflict, we performed experiments with DNA-PKcs deficient V3 cells, and their DNA-PKcs complemented derivative cell line V3-YAC, in which the gene for the human DNA-PKcs had been reintroduced via a yeast artificial chromosome (YAC). In clonogenic assays comparing the 2 cell lines, the LD_50_ values for topoisomerase II poisons (etoposide and doxorubicin), and LD_50_ values for ionizing radiation were significantly lower for the V3 (DNA-PKcs deficient) cells compared to the DNA-PKcs proficient V3-YAC cells, with dose reduction factors (DRF) between 2.4–2.9 (Supplementary Figure 3). In contrast, the alkylating agent 4-hydroperoxy-cyclophosphamide, the topoisomerase I poison topotecan and the platinum drug cisplatin did not show any significant differences in their LD_50_ values (Supplementary Figure 3). Temozolomide was slightly more cytotoxic in DNA-PK deficient V3 cells, but the DRF was only 1.6. For these reasons, chemopotentiation by NU7441 of doxorubicin and etoposide only was investigated further.

### Inhibition of DNA-PKcs by NU7441

Since DNA-PKcs expression and activity could potentially impact on drug- and radiosensitivity, these parameters were assessed by Western Blot. TC-71 and VH-64 had similar levels of DNA-PKcs protein (total DNA-PK) (Figure 1C). After DSB induction by ionizing radiation (10 Gy), there was a strong increase in signal intensity of phosphorylated DNA-PK at serine2056; a marker for DNA-PK activation. In both cell lines, co-treatment of the cells with NU7441 revealed inhibition of DNA-PK activity in a concentration dependent manner, with a maximal inhibition at 1.0 µM NU7441 in both cell lines (Figure 1D). All subsequent experiments were performed in the presence of 1.0 µM NU7441.

### Chemo- and radio-potentiation using the DNA-PK inhibitor NU7441

Using standard clonogenic assays on TC-71 and VH-64 cells, NU7441 alone was not toxic at 1.0 μM. In TC-71 cells, the NU7441 LD_50_ was 12 μM (*n* = 5 clonogenic assays, Figure 5A, left panel), and in VH-64 cells the LD_50_ was 16 μM (*n* = 1 clonogenic assay).

As predicted from the data presented in Supplementary Figure 3, NU7441 markedly potentiated the cytotoxicity of doxorubicin, etoposide and ionizing radiation when used at 1 μM (Figure 5A). NU7441 enhanced the cytotoxicity of doxorubicin 2-fold at the LD_50_ concentration (doxorubicin alone: LD_50 =_ 12–13 nM; doxorubicin with 1 μM NU7441 LD_50_ = 6 nM), and for both cell lines this effect was highly significant (2-way ANOVA *p =* 0.0008 for TC-71, *p* < 0.0001 for VH-64 cells). Potentiation of etoposide cytotoxicity by NU7441 was even greater (3–7 fold), with LD_50_ values for etoposide alone being 0.1 μM in VH-64 and 0.28 μM in TC-71 cells, reduced to 0.04 μM by NU7441, the effect again being highly significant in both cell lines (2-way ANOVA *p* < 0.0001). In addition, NU7441 potentiated the effects of ionizing radiation in both cell lines, causing a 3-fold reduction in cell survival (ionizing radiation alone LD_50_ = 1.0–1.3 Gy, ionizing radiation in presence of NU7441 LD_50_ = 0.36–0.4 Gy; 2-way ANOVA *p* < 0.0001). All of the NU7441 combination data are summarized in Table 1.

## DISCUSSION

Dysregulation of the DNA damage response has emerged over the past decade as both a contributor to genomic instability and thus carcinogenesis, but also as a possible therapeutic opportunity, either for overcoming drug resistance or for exploiting synthetic lethality [6]. This study evaluated inhibitors of two different DNA repair pathways, i.e. base excision repair (BER)/single strand break repair (SSBR) and non homologous end joining (NHEJ), representing the pathways responsible for DNA single and double strand break repair. Ewing sarcoma was investigated as there is an urgent clinical need to improve treatment outcomes in inoperable, metastatic and relapsed patients. Pilot data in DNA-PKcs deficient and proficient cells demonstrated that sensitivity to both ionizing radiation and drugs commonly used in treatment strategies for Ewing sarcoma (doxorubicin, etoposide) was greater in the DNA-PKcs deficient cells (Supplementary Figure 1).

Inhibitors of DNA-PKcs have been used *in vitro* in colon cancer models and CLL blasts to enhance sensitivity to radio- and chemo-therapeutic treatments [9, 10], but pediatric malignancies so far have not been studied. The inhibitor NU7441 has improved potency and specificity over its predecessors NU7026 and LY294002, with an IC_50_ of 14 nM and a > 100 fold specificity for DNA-PK over other PI3 kinase family members [22]. The expression and activity of DNA-PKcs was confirmed in ES cell lines, and concentration dependent inhibition by NU7441 was demonstrated. In *in vitro* assays, NU7441 was shown to sensitize ES cells to etoposide, doxorubicin and ionizing radiation with DRF_50_ values of 2.7–6.7, 2.1–2.2 and 2.8–3.4, respectively, all combinations being significantly different to the respective DNA-damaging agent alone in 2-sided ANOVA analyses. These are encouraging results that warrant further investigations in *in vivo* models. There are currently two DNA-PK inhibitors (MSC2490484A: DNA-PK inhibitor, trials NCT02316197 and NCT02516813; CC-115: dual DNA-PK and TOR kinase inhibitor, trial NCT01353625) undergoing testing in Phase I clinical trials in adult patients, including those with Ewing sarcoma, as single agents and for MSC2490484A also as a radio-/chemo-sensitizing agent.

PARP inhibition as an approach to the treatment of Ewing sarcoma has attracted increasing attention over the past years, after the publication by Garnett and colleagues identified the translocation EWS-FLI1 as a biomarker for PARP inhibitor sensitivity, and Brenner *et al.* reported that EWS-FLI1 interacts with PARP1 and influences its transcriptional activity. In other cancers, for example BRCA-deficient breast or ovarian cancer, sensitivity to PARP inhibition is due to defects in HRR, resulting in synthetic lethality. As EWS-FLI1 is reported to interact with BARD1, which associates with BRCA1, the HRR status of the ES cells was evaluated. Fluorescent microscopy assays for γH2AX and RAD51 foci clearly demonstrated that both ES cell lines were competent for HRR.

Evaluation of the PARP1 inhibitor rucaparib in the two ES cell lines confirmed previous publications by Brenner and Garnett that ES cells are sensitive to single agent PARP1 inhibitors. However, *in vitro* sensitivity was strongly influenced by the type of assay used, i.e. there was a 10-fold difference in LD_50_ depending on the duration of exposure to rucaparib (24 h standard assay *versus* continuous exposure). In our *in vivo* experiments, mice were treated with single agent rucaparib at the dose of 10 mg/kg i.p. daily for 5 days/week for the duration of the experiment. This dose and schedule previously led to delayed tumor growth in various BRCA deficient *in vivo* models [23] but did not show any measurable effect on ES tumor growth. Despite the dose used in mice being well below the RP2D for rucaparib monotherapy in ovarian cancer (600 mg, resulting in a C_max_ of 6 µM/l, equivalent to 100mg/kg in mice [24, 25]), this result however confirms *in vivo* studies of other PARP inhibitors (BMN673, olaparib) performed by Norris and by Smith from the Pediatric Preclinical Testing Program, who also failed to show any significant responses or survival benefit by single agent PARP inhibitors [26, 27]. Furthermore, a Phase II clinical trial with olaparib in patients with recurred/progressive ESFT did not report any clinical responses [28]. It is therefore likely that other, as yet unknown, factors render Ewing sarcoma tumors insensitive to single agent PARP inhibition *in vivo*.

In combination with DNA-damaging agents, rucaparib was able to sensitize ES cells to the effects of temozolomide, camptothecin and ionizing radiation, with the chemo-potentiation of temozolomide being the most profound (mean DRF_50_ 15–29). These results are in line with other published findings on chemo- and radio-potentiation both *in vitro* and *in vivo* ([29] and reviewed in [14]). Combinations of PARP inhibitors with chemotherapy (temozolomide or irinotecan, or both) in patients to date are still ongoing (e.g. NCT01858168, NCT02392793); however, systemic toxicity, especially myelosuppression, is anticipated.

Radio-potentiation via PARP- or alternatively DNA-PK-inhibition potentially bears valuable clinical benefit, as it would lack systemic toxicity and could improve the outcome for inoperable, large or axial Ewing sarcomas. Lee *et al.* have recently published results on radio-potentiation of ES cell lines by olaparib, both *in vitro* and using an *in vivo* preclinical model, with very encouraging results [30].

In summary, targeting DNA-repair pathways in combination with DNA-damaging agents is a promising approach to improving treatment strategies for Ewing sarcoma. Whereas inhibition of NHEJ by targeting DNA-PKcs needs further preclinical investigation *in vivo*, PARP inhibitors as chemo-sensitizers for temozolomide and irinotecan are already being investigated in clinical trials for Ewing sarcoma and other cancers. Results of these trials are eagerly awaited, and ionizing radiation also needs to be investigated in combination with these PARP inhibitors, for the benefit of patients with metastatic, inoperable or relapsed Ewing sarcoma.

## MATERIALS AND METHODS

### Chemicals

Temozolomide was a gift from Cancer Research UK, London, United Kingdom. Doxorubicin, etoposide and camptothecin were purchased from Sigma Aldrich, UK. The DNA-PK inhibitor NU7441 was synthesized at the Northern Institute for Cancer Research as described previously [22] and dissolved at 2 mM in DMSO. The PARP-inhibitor rucaparib (formerly AG014699) was a gift from Clovis Oncology, Inc. (Boulder, CO, USA) and dissolved in 10 mM DMSO.

### Cell lines and culture

The Chinese hamster ovary cell line V3 (deficient for DNA-PKcs) and its derivative cell line V3-YAC (transfected with the human gene for DNA-PKcs via a yeast artificial chromosome (YAC)) were a gift from Dr P. Jeggo (University of Sussex, UK).

The ES cell lines TC-71 [31] and VH-64 [32] with the typical translocation t(11;22)(q24;q12) resulting in the EWS/FLI-1 fusion transcript [33] were obtained as a gift from the department of pediatric hematology/oncology (Prof H. Jürgens University Hospital Münster, Germany). CADO-ES-1 cells [34] carrying the translocation t(21;22) (q22;q12) and leading to an EWS/ERG fusion transcript [35] were purchased from DSMZ (Germany). V3, V3-YAC and all ES cell lines were cultured in RPMI 1640 medium (Sigma-Aldrich, UK) containing 10% (v/v) fetal bovine serum (FBS), penicillin (100 U/ml) and streptomycin (0.1 mg/ml) at 37°C in a humidified atmosphere with 5% CO_2_ in air. The medium for ES cells contained additional 2 mM L-glutamine. V3-YAC cells were cultured in the presence of the antibiotic G-418 sulfate (500 μg/ml, Invitrogen, California, USA) to retain the YAC.

The human BRCA2 defective pancreatic carcinoma cell line CAPAN-1 was obtained from ATCC (Manassas, VA, USA) and maintained in RPMI 1640 medium with 15% (v/v) FBS [36]. The human breast cancer cell line MCF-7 and the hepatocellular carcinoma cell line Hep3B were obtained from the ATCC (Manassas, VA, USA), and both are HRR competent [23, 37]. MCF-7 cells were grown in the same medium as the ES cell lines, but without additional glutamine. Hep3B cells were cultured in DMEM/Ham’s F12 supplemented with 10% (v/v) FBS, 2 mM L-glutamine, penicillin (100 U/ml) and streptomycin (0.1 mg/ml). All cell lines were regularly confirmed mycoplasma free (MycoAlert^™^, Lonza) and used up to the 30th passage. Capan-1, Hep3B, MCF-7, VH-64 and TC-71 cells were authenticated by short tandem repeat profiling (LGC Standards) during the experimental work. All flasks and dishes intended for growing ES cells were collagen coated. For collagen coating, dishes were covered with a solution of rat tail collagen type I (BD Biosciences, 0.2 mg/ml in 0.1 M glacial acetic acid) and allowed to dry overnight in a tissue culture hood.

### Growth inhibition assays

Cell growth inhibition assays of exponentially growing TC-71, CADO-ES-1 and MCF-7 cells were performed in 6-well plates. Cells were seeded at a density of 1 × 10^4^ cells per well to ensure exponential growth for the duration of the assay. Twenty-four hours post seeding, cells were exposed to medium containing varying concentrations of temozolomide or camptothecin, in the presence or absence of 0.4 µM rucaparib in a final concentration of 0.5% (v/v) DMSO. The concentration of 0.4 µM was chosen as it had previously shown to enhance temozolomide and topotecan cytotoxicity in adult tumor cell lines [38]. In addition, cells were incubated with medium containing 0.4 µM rucaparib, and 0.5–1 h later exposed to varying doses of ionizing radiation (Gulmay Medical RS320 Irradiation System, Gulmay Medical Limited, Surrey, UK). Controls were 0.5% DMSO or 0.4 µM rucaparib alone as appropriate. Cells were harvested by trypsinisation 72 h later and counted using a CoulterCounter (Beckman coulter UK Ltd.). Cell growth as a percentage of the DMSO or rucaparib alone controls was plotted using GraphPad Prism software (Version 6, GraphPad Software, La Jolla, USA). Concentrations of cytotoxic drugs alone or in combination with rucaparib that inhibited growth by 50% (GI_50_) were calculated using GraphPad Prism software.

### Cell survival/cytotoxicity assays

Cell survival was determined by colony formation assays using one of 3 different procedures as follows: i) standard assay: exponentially growing cells were seeded onto 6-well plates or 6-cm petri dishes and 24 h later cells were exposed to drugs (temozolomide, camptothecin, doxorubicin, etoposide) with or without rucaparib (0.4 µM ) or NU7441 (1.0 µM) or the inhibitor alone for 24 h (0.4–50 µM), or exposed to ionizing radiation (2.69 Gy/min at 230 kV, 10 mA, Gulmay Medical Ltd., Surrey, UK), in the presence or absence of rucaparib (0.4 µM ) or NU7441 (1.0 µM), and incubated for a further 24 h +/− rucaparib/NU7441 as indicated. Cells were then harvested and re-seeded for colony formation in 10 cm dishes as previously described [23]. Cells treated with medium containing 0.5% (v/v) DMSO or inhibitor only (rucaparib 0.4 µM, NU7441 1.0 µM) were used as controls. ii) Cells were plated at low densities (50–1000) into 6-well dishes, and without harvesting or reseeding were either exposed continuously to different concentrations of rucaparib (0.04–50 µM) for the duration of the experiment (with the addition of fresh drug every 3–5 days); or iii) cells growing in 6-well dishes were exposed to rucaparib (0.4–50 µM) for 24 h followed by drug-free medium for the rest of the experiment. The usual assay performed was the standard assay as in i), only for rucaparib single agent assays were additional experiments (i.e. ii and iii) performed.

After 12–14 days at 37°C, colonies were stained with 0.4% (w/v) crystal violet and counted using an automated colony counter (ColCount, Oxford Optronics Ltd., Oxford, UK). As the VH-64 cells did not form satisfactory colonies on standard collagen-coated dishes, they were seeded into 6-cm Petri dishes containing a 0.16% (w/v) agarose-medium mixture (SeaKem ME Agarose, Lonza, Cologne, Germany) and stained after incubation for 12 days using 0.5 mg/ml MTT (Sigma, UK) for 2–5 h at 37°C. The cloning efficiency (%) was calculated as [(number of colonies counted/number of cells seeded) × 100] and cell survival/colony formation (%) was calculated as [(drug-treated cell cloning efficiency/control cell cloning efficiency) × 100]. The concentration of drug necessary to inhibit colony formation by 50% (LD_50_) was calculated, and the ratios of LD_50_ results for the cells treated with cytotoxic alone and the corresponding cells treated with cytotoxic plus inhibitor gave the dimensionless dose reduction factor_50_ (DRF_50_). Cells were plated in triplicates for each drug concentration and each chemo- or radio-potentiation experiment was repeated at least 2 times.

### Western blotting

Western blots for PARP1 were performed using the anti-PARP1 C2–10 primary antibody (Trevigen, MD, USA), as described previously [21].

To measure DNA-PK activity and its inhibition as well as total cellular DNA-PKcs protein levels, cells were either left un-irradiated or exposed to 10 Gy in the presence or absence of NU7441 (0.1–5.0 µM) and protein extraction was performed as previously described [39]. Briefly, after incubating for 30 minutes cellular proteins were extracted in Phosphosafe extraction reagent (Merck, UK), subjected to electrophoresis on 3–8% (w/w) Tris-Acetate XT-Criterion Gels (Biorad, UK), transferred onto nitrocellulose membranes (Hybond C, Amersham Biosciences, UK) and probed with mouse monoclonal DNA-PKcs (ab1832, Abcam, Cambridge, UK), rabbit polyclonal anti-pSer2056 DNA-PKcs (ab18192, Abcam, Cambridge, UK) and anti-Actin (Ab-1) mouse monoclonal antibody (JLA20) (Calbiochem, Nottingham, UK). Following exposure to HRP conjugated secondary antibodies and ECL development, expression was measured by chemiluminescence detection (Fuji LAS; Raytek, Sheffield UK).

### PARP activity assay

PARP activity and its inhibition was measured by a GCLP-validated immunoblot assay that has been previously described, both for clinical material and cell cultures [12, 40]. This assay measures poly ADP-ribose (PAR) formation in permeabilised cells following maximal stimulation by blunt ended oligonucleotides, mimicking DNA breaks, in the presence of excess NAD+. PAR formation was measured using anti-PAR 10H antibody (kind gift from Prof Dr A Burkle, University of Konstanz, Germany) followed by chemiluminescence detection as described above.

### Double strand break (DSB) induction and repair

DSB were counted by measuring phosphorylation of histone H2AX (γH2AX) by DNA-PK and ATM, leading to the formation of a γH2AX focus at the site of the lesion [41]. Rucaparib leads to the formation of DSB by inhibiting SSBR [23]. Measurement of nuclear RAD51 focus formation was used as a marker for functional HRR [42].

For the combined assay, TC-71, CADO-ES1 or Hep3B cells as an HRR competent positive control cell line were grown on round 22 mm collagen coated (for TC-71 and CADO-ES1) glass coverslips (BD Biosciences, UK) in standard 6-well plates for at least 24 h. Cells were then exposed to 10 μM rucaparib or vehicle control (i.e. DMSO) for 24 h at 37°C, washed twice with cold PBS, fixed with ice-cold 4% paraformaldehyde and probed for γH2AX and RAD51 using immunofluorescence microscopy with image analysis using ImageJ as previously described [23, 43]. Foci in 60-289 cells were counted for each cell line and treatment, on average 166 cells per experiment.

### Determination of *in vivo* anticancer activity

All animal experiments were performed according to current UK Home Office regulations, complying with the 3R principles (Home Office licence number PPL60/3846). Immunocompromised male rag2^–/–^γc^–/–^ mice were used and implanted intrafemorally with 5 × 10^5^ transduced TC-71 cells as previously described [44]. The TC-71 cells had been transduced with a lentiviral vector encoding both enhanced green fluorescent protein (EGFP) and firefly luciferase (fLuc) allowing to image growing tumors with bioluminescent imaging. Animals were randomly assigned to treatment with vehicle control (dH_2_O, 10 ml/kg i.p.) or with rucaparib 10 mg/kg i.p. on days 1–5 on a 7-day cycle for a duration of 6 cycles. The dose of 10 mg/kg i.p. daily x5 for 6 cycles was selected as it had been well tolerated previously and had shown significant delays in tumor growth in a BRCA1 and BRCA2 mutated mouse models [23]. Treatment of mice started on day 9 after intrafemoral injection of tumor cells, followed by weekly bioluminescent imaging as previously described [44].

### Statistical analyses

All graphs were plotted with the help of Graph Pad Prism software and statistical tests (2-way ANOVA) were calculated using GraphPad Prism (version 6.0).

## SUPPLEMENTARY MATERIALS FIGURES



## Abbreviations

ATCC: American Type Culture Collection
BER: Base excision repair
BRCA: Breast Cancer
CLL: Chronic lymphocytic leukemia
DMEM: Dulbecco’s Modified Eagle’s Medium
DMSO: Dimethylsulfonic acid
DNA: Deoxyribonucleic acid
DNAPK: DNA dependent protein kinase
DNA-PKcs: DNA dependent protein kinase catalytic subunit
DRF_50_ Dose reduction factor: ratio of two LD_50_ results (cells treated with cytotoxic alone and corresponding cells treated with cytotoxic plus inhibitor)
DSB: DNA-double strand breaks
γH2AX: phosphorylated histone H2AX
EFS: Event-free survival
e.g.: exempli gratia
EGFP: enhanced green fluorescent protein
ES: Ewing sarcoma
ESFT: Ewing sarcoma family of tumors
FBS: Fetal bovine serum
fLuc: firefly Luciferase
GI_50_ Growth inhibitory dose 50: Concentration that inhibits growth by 50%
Gy: Gray
H: hours
HRP: Horseraddish peroxidase
HRR: Homologous recombination repair
i.e.: id est
i.p.: intra peritoneal
LD_50_ Lethal dose 50: The concentration of drug necessary to inhibit colony formation by 50%
nM: nanomolar
mM: millimolar
µM: micromolar
MTT: 3-(4, 5-Dimethylthiazol-2-yl)-2,5-Diphenyltetrazolium Bromide
NAD: Nicotinamide-adenine dinucleotide
NHEJ: Non-homologous end joining
PAR: poly ADP-ribose
PARP: poly (ADP-ribose) polymerase
PBS: Phosphate buffered saline
RAD51: DNA repair protein
RP2D: Recommended phase two dose
RPMI: *Roswell Park Memorial Institute* medium
SD: Standard deviation
SEM: Standard error of mean
SSBR: Single strand break repair
YAC: Yeast artificial chromosome
