# Supplementary material for: Sensitizing Ewing sarcoma to chemo- and radiotherapy by inhibition of the DNA-repair enzymes DNA protein kinase (DNA-PK) and poly-ADP-ribose polymerase (PARP) 1/2
Source: Oncotarget. 2017 Sep 28;8(69):113418–30. doi: 10.18632/oncotarget.21300 (PMC5768336; doi:10.18632/oncotarget.21300)
Supplement: Supplementary file 1 [file oncotarget-08-113418-s001.pdf]

Sensitizing Ewing sarcoma to chemo- and radiotherapy by inhibition of the DNA-repair enzymes DNA protein kinase (DNA-PK) and poly-ADP-ribose polymerase (PARP) 1/2

SUPPLEMENTARY MATERIALS

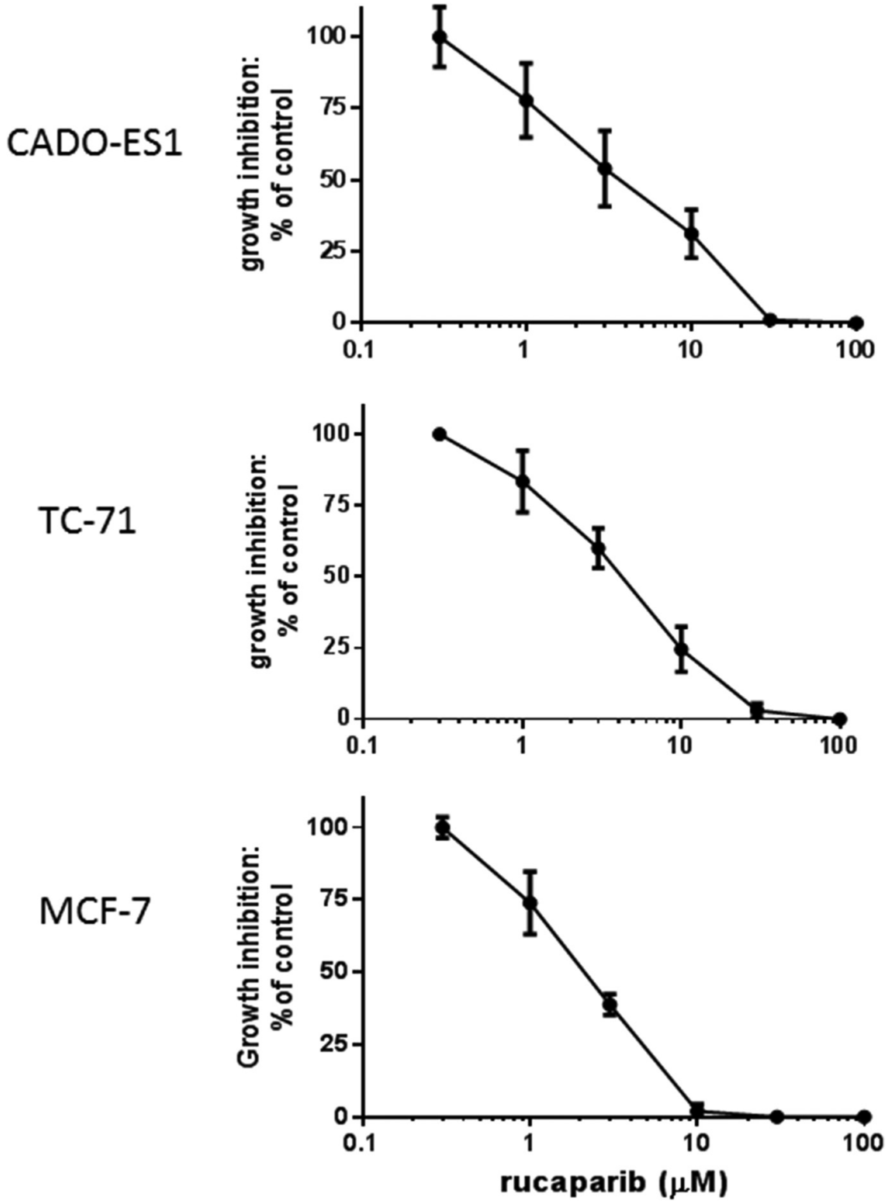

Supplementary Figure 1: Growth inhibition assays for rucaparib alone. Growth inhibition studies of rucaparib as a single agent in Cado-ES1, TC-71 and MCF7 cells. Points, mean of duplicate samples from two or three independent experiments; bars, SD.

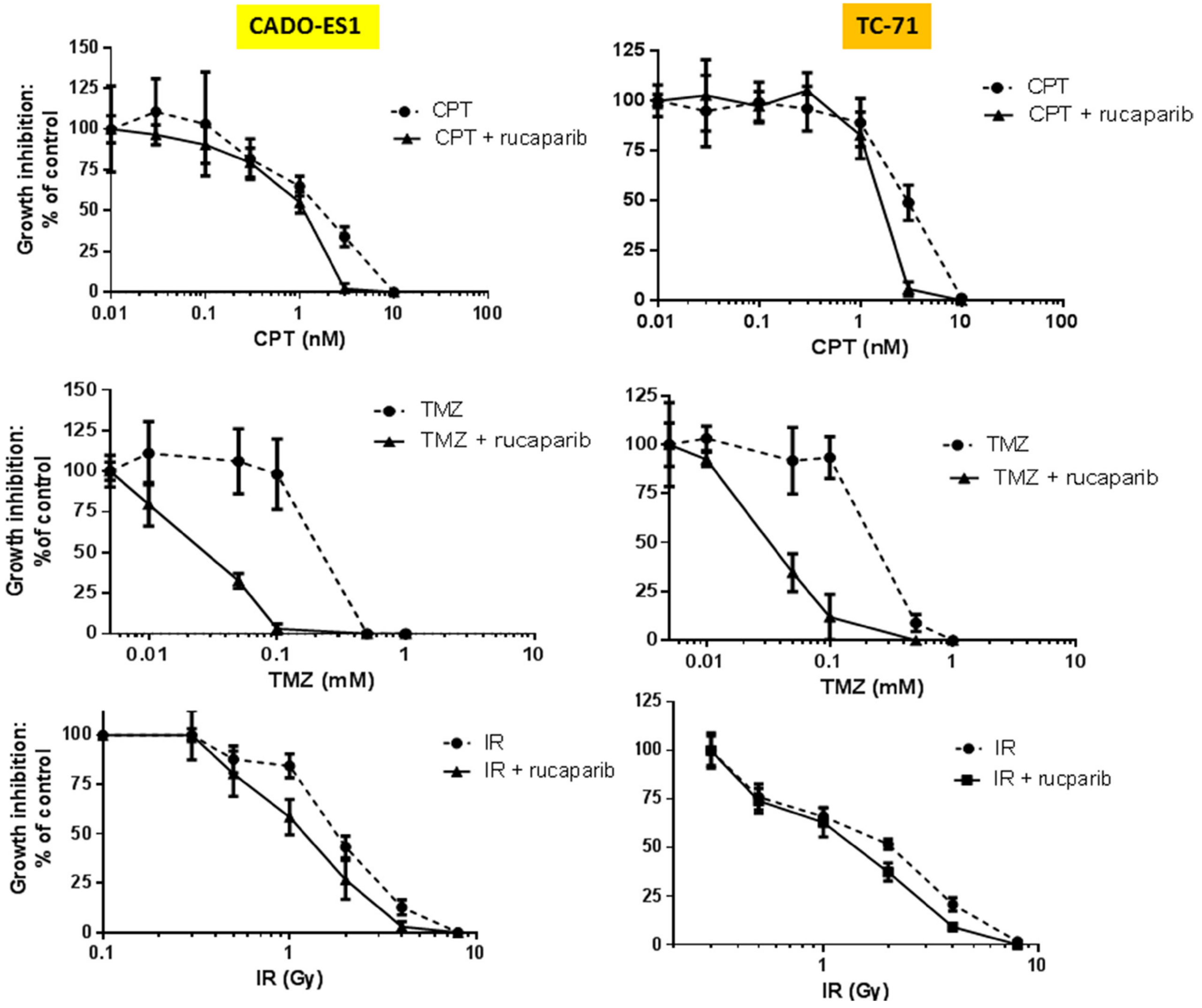

Supplementary Figure 2: Growth inhibition assays for rucaparib in combination with camptothecin, temozolomide or ionizing radiation. Growth inhibition studies of CADO-ES1 and TC-71 cells treated with camptothecin (CPT), temozolomide (TMZ) and ionizing radiation (IR) in the presence or absence of 0.4 μM rucaparib. Points, mean of duplicate samples of 2 or 3 independent experiments; bars, SD.

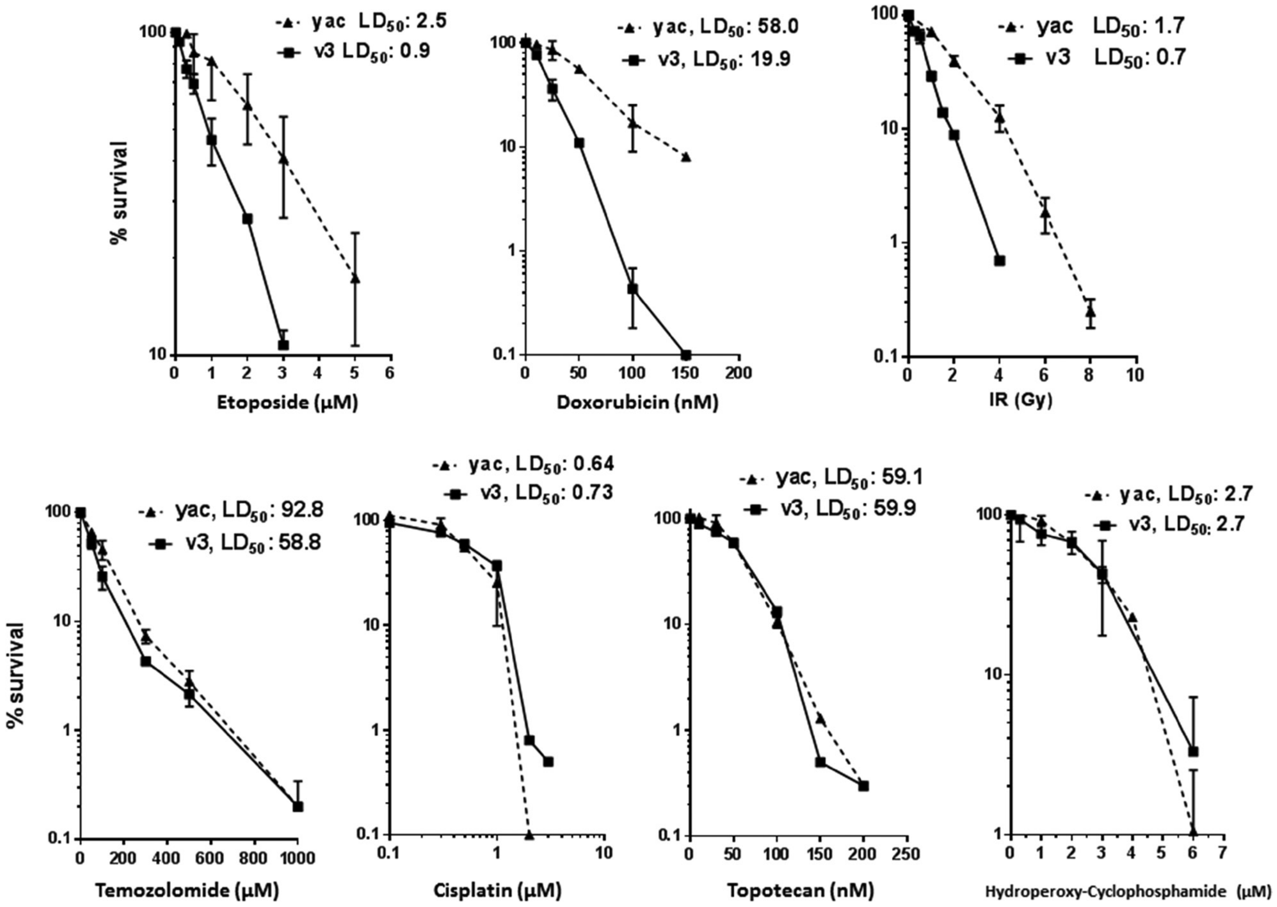

Supplementary Figure 3: Summary of clonogenic assays in a DNA-PK proficient and deficient cell line pair. Clonogenic assays in V3 (DNA-PKcs deficient) and V3-YAC (DNA-PKcs proficient) cells with different chemotherapeutic drugs. LD_{50}= lethal dose at which 50% reduction in colony formation is observed. Points, mean of triplicate samples from two to four independent experiments; bars, SD.
